# P-419. The Epidemiology of Healthcare-associated Infections in Jordanian Hospitals before and after the COVID-19 Pandemic

**DOI:** 10.1093/ofid/ofae631.620

**Published:** 2025-01-29

**Authors:** Salwa A Nasrat, Mohammad Alhawarat, Mohammad Gharaibeh, Omar Sayyouh, Mohamed Ramadan, Mayar Said, Tamer Saied Osman

**Affiliations:** NAMRU-EURAFCENT, Nasr City, Al Qahirah, Egypt; Ministry of Health, Jordan, Amman, 'Amman, Jordan; Ministry of Health, Jordan, Amman, 'Amman, Jordan; NAMRU-EURAFCENT, Nasr City, Al Qahirah, Egypt; NAMRU_EURAFCENT, Cairo, Al Qahirah, Egypt; NAMRU_EURAFCENT, Cairo, Al Qahirah, Egypt; NAMRU_EURAFCENT, Cairo, Al Qahirah, Egypt

## Abstract

**Background:**

The pandemic of COVID-19 resulted in an exceptional stress on the healthcare system. On the other hand, infection control measures were strengthened within the hospital settings. However, the effect on healthcare associated infections (HAIs) is still not clear, and data are still lacking. This study examines the rates of three HAIs [bloodstream infections (BSIs), Urinary tract infections (UTIs), and pneumonia] before the COVID-19 pandemics and compare them with corresponding rates after the pandemic.

**Methods:**

HAI data were extracted from the national surveillance of HAIs conducted by the Ministry of Health in Jordan. Data for 18 ICUs in 3 tertiary care hospitals were extracted and analyzed for this objective. An active prospective surveillance of HAIs was implemented using US CDC NHSN case definitions. Pooled incidence rates of three HAIs during the pre-pandemic (July 2018–March 2020) and post-pandemic periods (June 2021–December 2023) were calculated and compared.

**Results:**

During the study period, a total of 1642 HAIs were identified in 1624 patients for 302983 patient days. Male gender constituted 59.8% (412/689) among pre-pandemic patients compared to 61.6% (576/935) among post-pandemic patients (p value =0.4). Mean age of pre-pandemic patients was 44.68±1.12 years compared to 49.51± 0.88 years in the post-pandemic patients (p value =0.15). Specific HAI rates were significantly reduced after the pandemic compared to pre-pandemic period. Pooled pneumonia rate was reduced from 2.5/1000 patient-days to 1.4/1000 patient days (RR: 0.56; 95% CI, 0.47–0.67), UTI rate was reduced from 2.0/1000 patient days to 1.2/1000 patient days (RR: 0.59; 95% CI 0.49–0.71) and BSI rate from 2.6/1000 patient days to 2.6/1000 patient days to 2.0/1000 patient days (RR: 0.77; 95% CI 0.66–0.91). On the other hand, in hospital mortality rate for HAI patients significantly increased from 42.0% to 46.2% (p value =0.03).

**Conclusion:**

Post COVID-19 period was associated with a significant reduction in HAI rates. This could be due to increased compliance of the healthcare workers with the standards of infection control. Sustaining HAIs surveillance and auditing of infection control practices are needed to monitor HAIs rates and alert for waning compliance with infection control practices.

**Disclosures:**

**All Authors**: No reported disclosures

